# Uptake of measles second dose vaccine among children aged 15–35 months at Mettu Woreda, Illu Aba Bor Zone, Ethiopia: A community-based cross-sectional study

**DOI:** 10.1371/journal.pone.0342931

**Published:** 2026-02-12

**Authors:** Alemayehu Workiye Haile, Ebissa Bayana Kebede, Vinod Bagilkar, Teklu Wosenyeleh Mamo, Maycas Gembe

**Affiliations:** 1 Department of Nursing, College of Health Science, Mattu University, Mattu, Ethiopia; 2 School of Nursing, Institute of Health, Jimma University, Jimma, Ethiopia; 3 Department of Pediatric Nursing, Pattanshetti Institute of Medical Education and Research Centre’s, Seva Sadan School of Nursing, Gadhinglaj, Maharashtra, India; 4 Department of Epidemiology and Biostatistics, School of Public Health, Yekatit 12 Hospital Medical College, Addis Ababa, Ethiopia; Mizan-Tepi University, ETHIOPIA

## Abstract

**Background:**

Measles remains a significant global health threat, especially for Children. Its prevention can be achieved through a safe and affordable vaccine. However, there is a paucity of information about the uptake of the measles second-dose vaccine and associated factors in Ethiopia, particularly in Mettu Woreda. Furthermore, this study examined understudied variable maternal trust in healthcare workers.

**Objectives:**

To assess uptake of measles second dose vaccine and associated factors among children aged 15–35 months at Mettu woreda, Illu Aba bor zone, 2024.

**Methods:**

A community-based cross-sectional study was conducted from May to June of 2024 among 458 mothers/caregivers with Children aged 15–35 months using a systematic sampling technique. Data was collected using interviews, administered questionnaires. The Statistical Package for the Social Sciences software version 27 was used to analyze the data. Binary logistic regression was used to identify factors associated with the uptake of measles second dose vaccine at P-value < 0.05.

**Results:**

Coverage of the measles second dose vaccine was 58.5% (95% CI: 53.8–63.1). Factors positively associated with the uptake include maternal age (26−30 [AOR: 2.7, 95% CI (1.46–5.05)], 31−35 [AOR: 3.3, 95% CI (1.52–7.13)]), education; primary([AOR: 3.1, 95% CI (1.35–7.26)], secondary[AOR: 4.3, 95% CI (2.03–9.30)]), knowledge[AOR: 2, 95% CI (1.11–3.64)], information about MCV2[AOR: 3.7, 95% CI (2.10–6.56)], waiting time[AOR: 3, 95% CI (1.45–6.17)], while availability of vaccination service[AOR: 0.4, 95% CI (0.24–0.78)], and lack of trust in health workers[AOR: 0.3, 95% CI (0.18–0.55)] negatively affected vaccine uptake.

**Conclusion:**

The uptake of the measles second-dose vaccine among children aged 15–35 months is below district and national targets. Maternal age, education, knowledge, information access, trust in healthcare workers, antenatal care, waiting times, and service availability were all associated. Improved community awareness and addressing healthcare facility closures are needed.

## Introduction

Measles is a highly contagious vaccine-preventable viral disease that causes childhood morbidity and mortality in the World [1]. Measles has no particular therapy; however, the World Health Organization recommends two doses of vitamin A supplementations 24 hours apart for measles treatment and two doses of measles containing vaccines for measles prevention [2]. Measles vaccines are the safest and most effective public health measures available globally to prevent measles in children [3]. Ethiopia added the measles second dose vaccine (MCV2) to its routine immunization program on February 12, 2019 [4].

Measles outbreaks have continued to occur throughout the world, particularly in developing nations, despite the availability of safe and affordable vaccines [5]. According to the WHO and the United States Communicable Disease Control (CDC), 9 million measles cases occurred worldwide and resulting in 136,000 deaths, mostly in children [6]. Recently, measles outbreaks have continued in European countries, affecting children under the age of five who have not had vaccinations. As a result, 68% of children have been hospitalized due to measles-related complications like pneumonia and diarrhea [7].

Ethiopia recorded 16,814 laboratory-confirmed measles cases and 182 deaths between August 2021 and May 2023 [8].

In Ethiopia, the outbreak and its response had an overall economic cost of $758,869. Eighty percent of the overall economic cost was comprised health sector expenses, mostly from the immunization program, which cost $72.29 for each case. Ninety-two percent of the total expenses were covered by partner agencies [9].

The number of countries offering the measles second dose vaccine increased by 98% from 95 in 2000–188 in 2022; however, 11.3 million children remained unvaccinated in 2023 globally [10,11]. The estimated regional MCV2 coverage in the WHO Africa region rose from 7% in 2013 to 49% in 2023, which was substantial progress; however, it remains below the minimum levels needed to attain and maintain elimination [12].

Although the uptake of the second dose of the measles varies by Ethiopian district, there are constant trends concerning factors that are positively associated. These include maternal age, maternal knowledge, and participation in maternal health services (ANC visits, institutional delivery). For instance, A study in northwest Ethiopia found that the uptake measles second dose vaccine among under two years children was 53.08%. factors such as antenatal care visit, child delivery at health facility, and no long waiting time at the vaccination site positively associated with the uptake of measles second dose vaccine [13]. Similarly, a recent study in the Merhabete District of Central Ethiopia, reported the uptake of the measles second dose 63.3% and indicated that maternal knowledge, age, antenatal care service and institutional delivery positively associated with the uptake of measles second dose vaccine [14]. According a study done in the Nedjo District the uptake of measles second dose vaccine was 61.4% [15].

WHO launched the measles and rubella Strategic Framework (MRSF) 2021–2030, which adopts general structures of the immunization agenda 2030 (IA2030), aiming to eliminate measles and rubella from the world [16]. Ethiopia has implemented supplementary immunization activities (campaigns and reaching every district) strategies to attain at least 88% MCV2 coverage in each district and 93% MCV2 coverage nationally by 2025 [17]. Despite all of these efforts, measles outbreaks continued in many nations of the world [5]. There is a paucity of information on the coverage of the measles second dose vaccine; a recent systematic review highlighted a limited number of cross-sectional studies on MCV2 coverage and its associated factors in East African countries, resulting in a narrow evidence base [18].

Previously, some cross-sectional studies conducted in Ethiopia identified socio-demographic characteristics, utilization of maternal health services, and distance to health facilities as factors affecting MCV2 uptake [19–21]. However, to the best of our knowledge, important variables such as availability of vaccination service and mothers’/caregivers’ trust in health workers known to influence vaccine uptake in Kenya [22,23], which may hold significant influence in the Ethiopian context, were not yet studied in Ethiopia, particularly at Mettu Woreda, Illu Aba Bor zone, which borders zones that have experienced measles outbreaks [24,25]. Therefore, this community-based cross-sectional study, aimed to fill this knowledge gap by examining maternal trust in health workers. Assessing the MCV2 uptake and associated factors in this area is essential for effective prevention and control strategies.

## Methods and materials

### Study design, area and period

A community-based cross-sectional study was conducted at Mettu Woreda, Illu Aba Bora zone, Oromia region, Ethiopia, from May 13 to June 13, 2024. Mettu woreda is one of the woredas located in the Illu Aba Bor zone in the southwest part of Ethiopia. The woreda is located 600 km far away from Addis Ababa, the capital city of Ethiopia. It is bordered on the North by the West Wollega zone, on the West by Kelem Wollega, on the east by Buno Bedele, on the South by the Southwest Ethiopia region, and on the southwest by Gambella. According to the Ethiopian Central Statistical Agency population projection, the district has a total population of 88060 in 2023. Among the total population, 43835 are male and 44225 are female. There are 27 (26 rural kebeles and 1 urban kebele) kebeles in the woreda with a total of 18482 households. Among the total population, there are 14575 under-five children and 19631 reproductive-age women. Currently, the district has 27 health posts and four public Health centres that provide immunization services.

### Population

The study population for this study were Selected mothers/caregivers of children aged 15–35 months live in selected Kebeles. We included in the study all mothers/caregivers of children aged 15–35 months live in randomly selected kebeles for at least 6 months before data collection. While Mothers/caregivers who were unable to respond and Children whose appointment for measles second dose vaccine fall in the study period were excluded.

### Sample size determination

The sample size was estimated using a single population proportion formula; $n=\frac{{\left(Z_{\frac{a}{\text{2}}}\right)}^{\text{2}}P(\text{1}-P)}{d^{\text{2}}}.$ We consider the following assumptions: the standardized normal distribution curve (Zα/2) value for the 95% confidence interval = 1.96, P = 42.5% from the previous study [20] and margin of error (d) = 5%. $\text{n}=\frac{\left(\text{1}\text{9}\text{6}{)}^{\text{2}}\text{0}.\text{4}\text{2}\text{5}(\text{1}-\text{0}.\text{4}\text{2}\text{5}\right)}{{\left(\text{0}.\text{0}\text{5}\right)}^{\text{2}}}$ = 376. $\text{n}=\frac{n}{\text{1}+\text{n}/\text{N}}=\frac{\text{3}\text{7}\text{6}}{\text{1}+\text{3}\text{7}\text{6}/\text{1}\text{0}\text{5}\text{2}}=\text{2}\text{7}\text{7}$ After using the correction formula, since the total population is relatively small and to make feasible while maintaining level of statistical confidence considering the design effect of 1.5*277 = 416+and a 10% non-response rate, the final sample size was 416 + 41.6 = 458.

### Sampling technique and procedure

**Fig 1:**
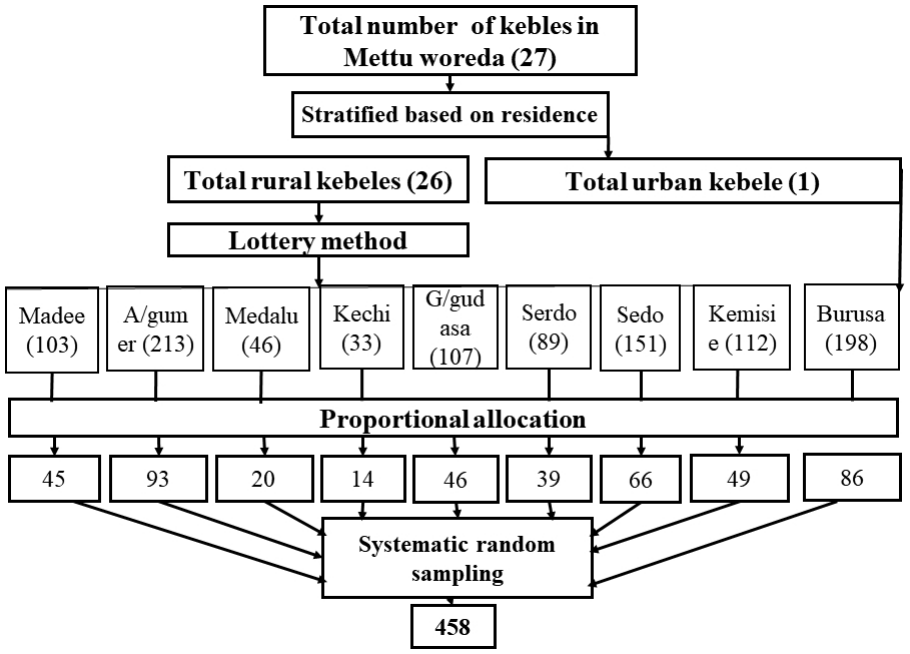
Diagrammatic presentation of sampling procedure and techniques.

First, the total number of kebeles in Mettu Woreda was stratified into rural and urban based on the residence. Then, eight rural Kebeles were selected using the lottery method, and the urban Kebele was taken to represent the urban population. The appropriate sample size was allocated for each selected Kebele based on the number of Households having children aged 15–35 months. A family folder was used as a sampling frame for the selection of households having children aged 15–35 months. Finally, individual households were selected by using a systematic random sampling technique. The sample interval (K = 2) in each household was obtained by dividing the total number of households by the allocated sample size. For those households having twins, one child was selected by the lottery method, and for those households having more than one eligible child, one child was selected by the lottery method. If the eligible participants were not present at home during data collection, data collectors revisited the household for a second time, and if they failed to be found after second visits, they considered it a non-response rate. The data collectors used roads, health posts, churches, mosques, and Kebele administrator offices as reference points to identify the selected Kebele border from the nearest Kebeles (Fig 1).

### Study variables

#### Dependent variable.

Uptake of measles second dose vaccine.

#### Independent variables.

**Socio-demographic characteristics of Mothers/caregivers** (age, education, residence, occupation, marital status, income, religion)

**Socio-demographic characteristics of the Child** (age, sex and birth order)

**Mothers/caregivers and obstetric history-related factors** (knowledge about measles immunization, media exposure, place of delivery, ANC and PNC services)

**Health system/service-related factors** (distance to Health facility, availability of a vaccine, waiting time for vaccination, information about MCV2, days of immunization, availability of service, mother/caregiver trust in the Health workers)

### Operational definitions

**Uptake:** the utilization of vaccination service for the measles second dose vaccine [20]. If a child had taken MCV2 between the ages of 15 and 35 months, he/she was considered to have received and coded as “1.” If he/she was not received coded as “0” [26].

**Caregiver:** the person who takes primary responsibility for the child who cannot take care of himself or herself fully, usually a family member [27].

**Good knowledge:** refers to those participants who scored greater than or equal to the mean of total knowledge score [19].

**Poor knowledge:** refers to those participants who scored less than the mean of total knowledge [19].

**Media exposure:** The mothers/caregivers were considered to have mass media exposure if they reported watching television or listening to the radio at least once a week and almost every day. The mothers/caregivers were considered to have used the internet if they reported using the internet every day or at least once a week in the past month [28].

**Trust in health workers:** If mothers/caregivers have moderate or very much trust in health workers who provide vaccines to children, they have trust in them, but if mothers/caregivers have little or no trust in health workers, they lack trust in them [29].

### Data collection tools and procedures

After reviewing different literature [19,20,22,29,30] interview-administered questionnaires were adapted. The questionnaire comprised sociodemographic characteristics of mothers/caregivers and children, obstetric history-related factors, and health system/service-related factors. A language expert translated the English version into Afan Oromo and Amharic and entered it into KoboToolbox. Four diploma nurses collected the data under the supervision of a supervisor (MSc in Nursing). The data was collected through observation of the child’s vaccination card for vaccination status and face-to-face interviews with mothers/caregivers for the sociodemographic characteristics of mother and child, mother’s/caregiver’s and obstetric-related factors, and health system-related factors. The data collectors asked the mother/caregiver to show the vaccination card of the child, and they entered the data into the Kobo toolbox. If the mother/caregiver could not show the vaccination card for various reasons, the data collectors asked the mother/caregiver to tell whether the child received MCV2 or not. To reduce recall bias, data collectors used different recall mechanisms such as the age of the child at which he/she received MCV2, the site of injection, and the number of MCV doses the child had taken after celebrating the 9^th^ month birthday.

### Data quality assurance

Before the actual data collection, orientation was given to data collectors and a supervisor for one day at Mettu town. The training focuses on the rules and regulations during data collection. In addition to these, the questionnaire was pretested for consistency on 5% of the total sample size (23 households) one week before the data collection date at Hurumu Woreda, which is outside of the study area. After the pretest, some corrections (order and flow of questions, adding options and adding skips) were made to the questionnaire as necessary. The validity was ensured by two experts. The supervisor and principal investigator checked the questionnaires daily to ensure completeness.

### Data processing and analysis

The collected data was exported from the Kobo toolbox to SPSS version 27, then it was cleaned and coded for data analysis. A binary logistic regression model was used to look at the statistical association between the outcome variable and every single independent variable. Variables that showed statistical significance during bivariable analysis at P-value < 0.25 were entered into multivariable logistic regression. The strength of associations was estimated by using adjusted odds ratios (AOR) with a 95% confidence interval, and significance was declared at a p-value < 0.05. Multi-collinearity was checked by using the Variance Inflation Factor (VIF). The model fitted the data adequately since the Hosmer-Lemeshow test was insignificant (P-value = 0.489). Analysis of the data was done using the forward stepwise logistic regression method. The results were presented using statements, tables and graphs.

### Ethical considerations

Ethical approval for this study was obtained from Jimma University, Institute of Health Institutional Research Board (IRB) by reference number JUIH/IRB/133/24. Then permission letter was written from Mettu Woreda to each kebele administrator and health post. After all participants were fully informed about the study’s purpose, potential benefits and their right to withdraw at any time, Written informed consent was obtained. Confidentiality and privacy were maintained throughout the study procedure.

## Results

### Socio-demographic characteristics of mothers and children

A total of 451 respondents participated in this study, giving a response rate of 98.4%. One hundred seventy-eight (39.5%) of the respondents were ≤25 years old. The participants had an average age of 27.5 years ± 4.9 years (Mean ± SD). In terms of their education, 135 (29.9%) of respondents were unable to read and write. 366 (81%) were rural dwellers (Table 1).

**Table 1:** Socio-demographic characteristics of mothers/caregivers and children aged 15-35 months at Mettu Woreda, Oromia, Ethiopia 2024. (N = 451).

Variable	Category	Frequency	Percentage
Age of mother/caregiver	≤25 years	178	39.5
	26-30 years	149	33
	31-35 years	86	19.1
	≥36 years	38	8.4
Religion of mother/caregiver	Orthodox	106	23.5
	Muslim	158	35
	Protestant	187	41.5
Marital status of the mother	Married	438	97.1
	Divorced	13	2.9
Respondent’s relationship to the child	Mother	443	98.2
	Grandmother	8	1.8
Sex of child	Male	203	45
	Female	248	55
Birth order	First	161	35.7
	Second	78	17.3
	Third	109	24.2
	Fourth and above	103	22.8
Residence	Rural	366	81.2
	Urban	85	18.8
Mothers’/caregivers’ education	Unable to read & write	135	29.9
	Able to read & write	96	21.3
	Primary school	89	19.8
	Secondary school & above	131	29
Mother’s occupation	Housewife	371	82.3
	Merchant	65	14.4
	Government employee	15	3.3
Family monthly income	<5000 ETB	231	51
	≥5000 ETB	220	49

### Mother/Caregiver and obstetrics history-related factors and uptake of MCV2

One hundred ninety-five (43.2%) of the respondents did not know about measles immunization, and more than one-third of them did not know the immunization schedule of the measles vaccine. Nearly three-fourths of mothers gave birth at health facilities. Among those participants, 231 (51.2%) had utilized postnatal care services. Of the respondents, only 97 (21.5%) attended four or above antenatal care services during the pregnancy of the index child (Tables 2 and 3).

**Table 2:** Distribution of mothers’/caregivers’ knowledge about measles vaccination at Mettu Woreda, Oromia, Ethiopia, 2024. (N = 451).

Variable	Category	Frequency	Percent
Know about measles immunization	Yes	256	56.8
	No	195	43.2
Recommended age to start measles vaccination	Just after birth	67	14.9
	1 month after birth	147	32.6
	At nine months	190	42.1
	I don’t know	47	10.4
Recommended MCV doses for children ≤ 2 years	One	46	10.2
	Two	243	53.9
	Three	60	13.3
	I don’t know	102	22.6
Know the measles vaccination schedule	Yes	292	64.7
	No	159	35.3
Keep measles vaccination appointment	Yes	203	45
	No	248	55
Immunize baby with all MCV doses	Yes	194	43
	No	257	57
Measles vaccine will make your child sick	Yes	216	48
	No	235	52
Knowledge	Good knowledge	273	61
	Poor knowledge	178	39

**Table 3:** Obstetrics history-related factors of mothers having children aged 15-35 months at Mettu Woreda, Oromia, Ethiopia, 2024. (N = 451).

Variable	Category	Frequency	Percent
ANC services	Yes	307	68
	No	144	32
No of ANC Visits	No ANC Visits	144	32
	1-3 ANC Visits	210	46.5
	≥4 ANC Visits	97	21.5
PNC services	Yes	231	51.2
	No	220	48.8
Place of delivery	Home	113	25
	Health facility	338	75

### Health system/health service related factors and uptake of MCV2

One hundred eighty-nine (41.9%) of the participants had not received information about MCV2 from health professionals, and 115 (25.5%) of the participants waited more than 30 minutes at the health facility to get the vaccination services. More than half (51.2%) of the participants had no trust in health workers (Table 4).

**Table 4:** Distribution of Health System/Health service-related factors among study participants at Mettu Woreda, Oromia, Ethiopia, 2024. (N = 451).

Variable	Category	Frequency	Percent
Got information about MCV2	Yes	262	58.1
	No	189	41.9
Availability of service	Yes	298	66
	No	153	34
Distance to the Health facility	<30 Minutes	302	67
	≥30 Minutes	149	33
Waiting time at Health facility	<30 Minutes	336	74.5
	≥30 Minutes	115	25.5
Lack of trust in Health workers	Yes	231	51.3
	No	220	48.8

### Magnitude of uptake of measles second dose vaccine (MCV2)

Of the total of 451 children included in this study, 264 (58.5%) (95% CI: 53.8–63.1) of them received the measles second dose vaccine (MCV2).

### Reasons for not receiving MCV2

**Fig 2:**
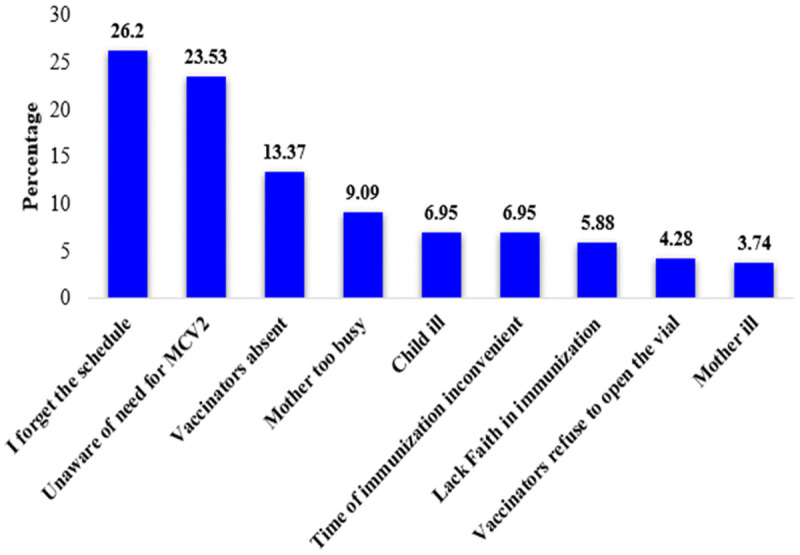
Reasons for not receiving measles second dose vaccine among respondents in Mettu Woreda, Ethiopia, 2024.

Out of 451 participants, 187(41.5%) didn’t vaccinate their children for MCV2. The most common reason cited by the participants for not vaccinating their children with MCV2 was forgetting the schedule, 49(26.2%) (Fig 2).

### Factors associated with the uptake of measles second dose vaccine (MCV2)

Both bivariable and multivariable logistic regression were performed to ascertain the effect of independent variables on the dependent variable. In bivariable binary logistic regression analysis, mothers’/caregivers’; age, educational level, knowledge about measles vaccination, monthly income, trust in healthcare workers, birth order of the child, sex of the child, place of delivery, postnatal care (PNC) and antenatal care (ANC) service, media exposure, information about MCV2, distance to nearest immunization center, waiting time to get a vaccination, and availability of service were associated with MCV2 uptake at P < 0.25. However, after adjusting for potential confounders in the multivariable logistic regression analysis, mothers’/caregivers’ age, educational level, knowledge about measles vaccination, trust in health workers, ANC service, information about MCV2, waiting time, and availability of service significantly associated with MCV2 uptake at P < 0.05.Accordingly, children whose mothers/caregivers were aged 26–30 years had 2.7 times higher odds of receiving MCV2 [, AOR: 2.795% CI (1.46–5.05)]. Similarly, children whose mothers/caregivers aged 31–35 and ≥36 years had 3.3 and 4 times higher odds of receive MCV2 than children whose mothers/caregivers aged ≤ 25 years [AOR: 3.3, 95% CI (1.52–7.13)] and [AOR: 4., 95% CI (1.25–12.79)]. The odds of receiving MCV2 were 3.1times higher for children whose mothers completed primary school and 4.3 times for children whose mothers completed secondary school or above than children whose mothers were unable to read and write [AOR: 3.1, 95% CI (1.35–7.26)] and [AOR: 4.3, 95% CI (2.03–9.30)].

The odds of receiving MCV2 for Children whose mothers/caregivers had good knowledge about measles vaccination were doubled [AOR: 2, 95% CI (1.11–3.64)]. The odds of receiving MCV2 were 3.1 and 4.4times higher for children whose mothers had antenatal care follow-up for 1–3 and ≥4 times [AOR: 3.1, 95% CI (1.62–6.05)] and [AOR: 4.4, 95% CI (1.83–10.85)], respectively, than children whose mothers did not have Antenatal care follow-up.

From Health System/Health service-related factors, findings revealed that mothers/caregivers who had been given information about MCV2 had 3.7 times higher odds to vaccinate their children than their counterparts[AOR: 3.7, 95% CI (2.10–6.56)]. The odds of receiving MCV2 were three times higher for children whose mothers/caregivers waited for ≤30 minutes at the health facility than children whose mothers waited >30 minutes [AOR: 3, 95% CI (1.45–6.17)]. Children whose mothers/caregivers got closed health facilities when they visited health facilities for vaccination service were 60% less likely to receive MCV2 than children whose mothers/caregivers got open health facilities [AOR: 0.4, 95% CI (0.24–0.78)]. Children whose mothers/caregivers had no trust in Health workers were 70% less likely to be vaccinated with MCV2 than children whose mothers/caregivers had trust in Health workers[AOR: 0.3, 95% CI (0.18–0.55)] (Table 5).

**Table 5:** Multivariable logistic regression analysis of factors affecting the uptake of MCV2 among children aged 15-35 months at Mettu Woreda, Oromia, Ethiopia, 2024. (n = 451).

Variable	Category	Uptake of MCV2		COR(95%CI)	AOR(95%CI)	P-value
		Received	Not Received			
Mother’s/caregiver’s age	≤25 Years	72	106	1	1	
	26-30 Years	100	49	3.01 (1.91-4.73)	2.7 (1.46-5.05)	0.002
	31-35 Years	61	25	3.59 (2.07-6.25)	3.3(1.52-7.13)	0.002
	≥36 Years	31	7	6.52 (2.72-15.61)	4 (1.25-12.7)	0.019
Mother’s/caregiver’s education	Unable to read & write	34	101	1	1	
	Primary school	75	14	15.91(7.98-31.74)	3.1 (1.35-7.26)	0.008
	Secondary & above	111	20	16.49(8.92-30.48)	4.3 (2.03-9.30)	0.001
Knowledge	Poor	55	123	1	1	
	Good	209	64	7.30 (4.78-11.16)	2 (1.11-3.64)	0.020
No of ANC Visits	No ANC visit	36	108	1	1	
	1-3 ANC Visits	147	63	7.00 (4.34-11.30)	3.1 (1.62-6.05)	0.001
	≥4 ANC Visits	81	16	15.19(7.88-29.26)	4.4 (1.83-10.8)	0.001
Got information about MCV2	No	86	103	1	1	
	Yes	178	84	2.54 (1.73-3.74)	3.7 (2.10-6.56)	0.001
Waiting time at Health facility	<30 Minutes	241	95	10.15(6.06-16.98)	3 (1.45-6.17)	0.003
	≥30 Minutes	23	92	1	1	
Availability of service	No	66	87	0.38 (0.26- 0.57)	0.4 (0.24-0.78)	0.006
	Yes	198	100	1	1	
Lack of trust in Health workers	No	166	54	1	1	
	Yes	98	133	0.24 (0.16-0.36)	0. 3 (0.18-0.55)	0.001

1 = Indicates a reference for the group.

## Discussion

The study aimed to assess the uptake of the second dose of the measles vaccine (MCV2) among children aged 15–35 months in Mettu Woreda. Our study finding revealed that 58.5% children were received MCV2. It can be concluded that maternal and facility related factors are important factors associated with the uptake of MCV2.

This study is lower than the global measles immunization target set by the WHO (≥95%) and the Ethiopia minister of health 88% MCV2 coverage [17]. This study is in line with studies conducted in Indonesia and Kenya, which revealed 54% and 56.2% respectively [31,32].

However, this study is lower than the studies carried out in China and Ghana, which revealed 95.8% and 82.8% of MCV2 coverage, respectively [33,34]. This discrepancy might be due to the early initiation of MCV2 vaccination in both Ghana and China, in addition to the large sample sizes used in the Chinese study. The studies were conducted 8 and 14 years after the MCV2 was launched in Ghana and China, respectively. This could give a chance to the community to raise their awareness about MCV2; on the other hand, the late initiation of the vaccination could influence mothers’ perceptions and willingness to accept the vaccine [21]. Furthermore, the disparity might be due to the data collection method and the socio-demographic characteristics of the study participants in Ghana and China, in which the vaccination status of the children was determined from the children’s vaccination cards. This could result in a lower or higher estimation of vaccination coverage. This study also lower than a study conducted in Gondar, Ethiopia, which revealed 75.68% [35]. These variations might be due to sociodemographic factors like participants’ educational attainment and residence; in the Gondar study, 69.53% of mothers attended secondary school and above, and the majority (90.98%) of them were urban dwellers, whereas, in this study, only 29% of mothers completed secondary school, and the majority (81.8%) of them were from rural areas. Other evidence indicated that vaccination rates were greater in urban areas than in rural areas [36]. This might be due to that mothers or caregivers who live in urban areas could have better access to information and health services.

The current study is higher than the Ethiopian mini demographic Health survey result of the Oromia region, which revealed 5.2% [36] and a cross-sectional study in the urban area of North Shoa, which found 42.5%. This might be due to the study period and sample size. The Ethiopian min Demographic Health survey was conducted at the time of the MCV2 introduction in Ethiopia’s routine immunization schedule [36]. Furthermore, this variation might be attributed to the COVID-19 pandemic, which might lead mothers and caregivers to be afraid to bring their children to healthcare facilities due to fears of exposure to the virus [37].

According to this study, mothers/caregivers aged above 25 years were more likely to vaccinate their children with MCV2 than those mothers/caregivers younger than 25 years. The study is supported by a study done in the North Shoa Zone, Ethiopia. However, this finding is contrary to another study done in Ethiopia, which found that children of older mothers were less likely to receive MCV2 than children of younger mothers [38]. This could be due to the study area (country-wide) and data collection method (secondary data analysis) used in that study. The fact that older mothers and caregivers may have more experience in utilizing healthcare services.

Mothers/caregivers who completed primary and secondary education or higher were more likely to vaccinate their children with MCV2 compared to mothers/caregivers who were unable to read and write. This study agrees with previous studies carried out in the Democratic Republic of Congo [39] and Northwest Ethiopia [19]. However, this study is inconsistent with a study done in Ghana which reported no statistical significance between maternal education and MCV2 uptake [33]. The variation might be due to that most (83.2%) of the study participants in the Ghana study had similar levels of education. Mothers with higher educational levels could recognize the value of vaccination and could obtain reliable vaccination information [21].

According to the current study, mothers/caregivers who had good knowledge about measles immunization were more likely to vaccinate their children for MCV2 than their counterparts. This finding is supported by studies done in Kenya [40], Ghana [33] and Ethiopia [19,20,26]. Similarly, children whose mothers had four or more ANC follow-ups were more likely to be vaccinated with MCV2 than those children whose mothers had no antenatal care follow-ups. This finding is in line with studies done in Ethiopia and Kenya [27,35].

The result of this study found that mothers/caregivers who got information about MCV2 from health professionals and waited for less than thirty minutes at the health facility to get the measles vaccine were more likely to vaccinate their children for MCV2 than their counterparts. These findings are consistent with studies done in Ethiopia and Kenya [19,40].

Long waiting times at the health facilities might interfere with mothers’/caregivers’ daily routine activities and discourage them from vaccinating their children. This study indicated that mothers/caregivers who got closed health facilities when they visited for vaccination were less likely to vaccinate their children compared to their counterparts. This finding agrees with a study in Mwingi Central Sub County [22], which indicated that the facility being opened always improves MCV2 uptake.

Mothers/caregivers who had no trust in health workers were less likely to vaccinate their children than those who had trust in health workers. This finding is supported by a study in Kenya [23], which revealed that mothers who trusted health workers were less likely to have a child with missed vaccine doses. This study has strengths and limitations. The inclusion of previously unstudied variables provides a more comprehensive understanding of measles second dose vaccine uptake in the study population, while the limitations are potential bias in self-reported vaccination history.

## Conclusion

This study found that the uptake of MCV2 among children aged 15–35 months at Mettu Woreda was lower than both the district and national MCV2 coverage targets set by the Ethiopian Ministry of Health. Factors influencing the uptake of the measles second dose vaccine include the mother’s or caregiver’s age and education, antenatal care visits, knowledge of immunization, information on MCV2, waiting time at health facilities, service availability, and trust in health workers. The findings highlight the importance of comprehensive interventions (enhancing maternal education, raising awareness, strengthening ANC services, and establishing trust with health workers) that target the factors that have been identified. Problems should be addressed that result in closed Health facilities on vaccination dates.
